# Face name matching and memory complaints in Parkinson’s disease

**DOI:** 10.3389/fpsyg.2022.1051488

**Published:** 2022-11-14

**Authors:** Antònia Siquier, Pilar Andrés

**Affiliations:** ^1^Neuropsychology and Cognition Research Group, Department of Psychology, Institute of Health Sciences (IUNICS), University of the Balearic Islands, Palma, Spain; ^2^Health Research Institute of the Balearic Islands (IdISBa), Palma, Spain

**Keywords:** Parkinson’s disease, associative memory, subjective memory complaints, neuropsychological tests, FNAME

## Abstract

**Objective:**

Memory impairment is a hallmark cognitive deficit in Parkinson’s disease (PD). However, it remains unclear which processes underlie this deficit in PD. Also, little is known on these patients’ subjective experiences of memory difficulties and their relationship with objective measures. We aim to portray memory deficits in PD by combining objective and subjective memory measures.

**Methods:**

Fifteen PD patients and 15 controls were assessed with an extended version of the Face-Name Associative Memory Exam (FNAME) and the Memory Failures of Everyday Questionnaire (MFE-28). We also explored the relationship among clinical and cognitive variables.

**Results:**

Participants with PD presented with more memory complaints. On the FNAME, these patients exhibited lower performance in free recall, as well as in name recognition and matching. Importantly, when controlling for initial learning, group effects disappeared, except for matching. Associative memory therefore was significantly compromised in PD and correlated with subjective memory complaints (SMC).

**Conclusion:**

Our findings suggest that associative memory may constitute a sensitive measure to detect subtle memory deficits in PD. Moreover, the current study further clarifies the source of memory impairment in PD. Thus, our study highlights the clinical value of including associative memory tests such as the FNAME in PD neuropsychological assessment.

## Introduction

Memory impairment represents a hallmark cognitive deficit in PD, even before the onset of motor symptoms in the prodromal states (Muslimović et al., 2005; Yarnall et al., 2014; Weintraub et al., 2015; Aarsland et al., 2017). Also, memory failures are among the most frequent cognitive complaints in PD, emerging as potential predictors of progression to a further decline or dementia (Erro et al., 2014; Pascual-Leone et al., 2018; Galtier et al., 2019; Gasca-Salas et al., 2020). The objective and subjective assessment of memory performance is therefore clinically important for the early identification of patients with a potential risk of future cognitive decline and, subsequently, for enabling treatment at the earliest possible stage.

Differences in memory functioning have been identified at retrieval and encoding stages. The first are attributable to a fronto-striatal dysfunction and appear to originate from an executive impairment. According to this, the pattern of performance of these patients is characterized by difficulties in free recall, whereas recognition and cued recall are thought to be relatively spared (Whittington et al., 2006; Costa et al., 2014; Economou et al., 2016). However, recognition and cued-recall deficits have also been observed (Carlesimo et al., 2012; Foo et al., 2016; Das et al., 2019; La et al., 2019). For instance, differences in recall performance between individuals with PD and healthy controls may disappear when encoding is equated across groups (Chiaravalloti et al., 2014; Siquier and Andrés, 2021a). Hence, evidence suggests that memory dysfunction in individuals with PD may reflect hippocampal alterations rather than solely fronto-striatal alterations. Neuroimaging studies provide support for this position by demonstrating associations between hippocampal alterations in PD and memory dysfunction (see Pourzinal et al., 2021 for a review).

Most studies on episodic memory in PD have used verbal or semantic stimuli (Baran et al., 2009; Hanoğlu et al., 2019; Siquier and Andrés, 2021a). However, one of the neuropsychological tests that has proven especially sensitive to detect subtle memory changes in early stages of the aging continuum, even in preclinical subjects (Rentz et al., 2011; Rubiño and Andrés, 2018; Sanabria et al., 2018), combines face and name stimuli. Face-name associations require multi-modal visual and verbal integration, a role that is thought to involve the hippocampal system (Werheid and Clare, 2007). Previous neuroimaging findings in PD have provided further support for using the associative memory paradigms as a marker of hippocampal dysfunction underlying associative memory deficits in PD (Cohn et al., 2016). In that sense, associative memory tasks appear to provide a valid measure of hippocampal function in PD.

The Face–Name Associative Memory Exam (Rentz et al., 2011) is an associative memory test requiring binding names and faces, of which a short version was developed by Papp et al. (2014), and subsequently adapted for the Dutch (Enriquez-Geppert et al., 2021), Spanish (Alegret et al., 2015a,^2020^; Flores Vazquez et al., 2021), Latino-American (Vila-Castelar et al., 2020), and Greek (Kormas et al., 2018) populations. This short version has exhibited an excellent convergent validity with the original test (Amariglio et al., 2012) and with other episodic memory tests such as the Free and Cued Selective Reminding Test (Papp et al., 2014; Vila-Castelar et al., 2020), the Auditory Verbal Learning Test and the Rey Osterrieth Complex Figure Test (Kormas et al., 2018), and The Word List Learning test from the Wechsler Memory Scale-Third Edition (WMS-III) (Alegret et al., 2015b). Recently, Enriquez-Geppert et al. (2021) explored age-related changes and showed clear aging effects on most recall measures. These results were replicated by Flores Vazquez et al. (2021) in Spanish and Mexican populations including two newly additional subtests to cover further memory subdomains and add clinical value: Spontaneous Name Delayed Recall, assessing free recall, and Face-Name Matching, assessing binding more specifically. These two subtests allow for examination of the extent to which memory deficits might result from a failure in binding processes related to hippocampal circuitries—in line with the arguably critical role of the hippocampal dysfunction in associative memory deficits in PD (Cohn et al., 2016).

There are different memory processes (i.e., immediate and delayed recall and recognition) that are assessed in the FNAME. In terms of analyzing the memory processes that can be affected in PD, one advantage of this test is that its task structure allows to control for encoding strategies during the initial learning phase. This control is important because subsequent retrieval is dependent upon learning acquisition. The FNAME therefore allows one to control for encoding memory deficits and distinguish them from retrieval deficits (improved by recognition, typical in dysexecutive syndrome).

An important issue in memory research is also the comparison between subjective and objective memory assessment. To date, few investigations have focused on the study of subjective memory decline beyond executive deficits in PD (Erro et al., 2014; Lehrner et al., 2014; Baschi et al., 2018; Gasca-Salas et al., 2020), and their results are inconclusive. For example, while Lehrner et al. (2014) observed significant associations in PD between objective and subjective memory measures, tested with the Verbal Selective Reminding Test (VSRT) and the Forgetfulness Assessment Inventory (FAI) scale, respectively, Erro et al. (2014) did not find a clear association between subjective memory complaints (SMC) and objective cognitive decline. This discrepancy may result from Erro et al.’s use of a single question to screen SMC (i.e., “Problems remembering things that have happened recently or forgetting to do things”). Importantly, however, both authors found that individuals with PD reported significantly more memory complaints than healthy controls. Interestingly, Gasca-Salas et al. (2020) reported a significant association between individuals with PD’ complaints about forgetting names and visuospatial impairment in these patients, while complaints did not correlate significantly with performance in other cognitive domains.

The low ecological validity of neuropsychological tests frequently used (Koerts et al., 2011; Vlagsma et al., 2017) may sometimes explain the absence of concordance between objective and subjective memory measures. By contrast, self-report questionnaires and associative face name measures may better capture the real-world symptomatology of these patients with respect to memory performance (Isquith et al., 2013; Polcher et al., 2017; Mariano et al., 2020).

The main aim of the present study was to investigate episodic memory deficits in individuals with PD and their subjective experience by using the FNAME^1^ to better understand memory difficulties in these patients. Furthermore, while the FNAME shows promise as an ecologically valid memory measure, little is currently known about its value as a correlate of everyday memory functioning. It would therefore be important to assess the extent to which performance on the FNAME correlates with SMC in patients with PD. Given the known involvement of the hippocampus in learning and associative memory (Cohn et al., 2016; Bezdicek et al., 2019; Pourzinal et al., 2022), we predicted that the PD group would show objective memory deficits, explained by encoding and retrieval mechanisms. We also predicted that individuals with PD would report greater memory difficulties in daily functioning, and that a negative correlation with the objective memory assessment obtained in the FNAME would be observed. Given the impact of memory deficits in PD and that SMC have been considered a predictor of future cognitive impairment in PD (Erro et al., 2014; Hong et al., 2018; Galtier et al., 2019; Gasca-Salas et al., 2020), the present study provides important information about the characterization of memory impairment in PD from objective and subjective angles.

## Materials and methods

### Participants

The present study is based on the same clinical sample previously reported in Siquier and Andrés (2021a,b, 2022). We recruited fifteen individuals with PD (one woman, age 67.4 ± 9.7) from the neurology Department of a tertiary hospital. All patients fulfilled the UK Brain Bank diagnostic criteria for PD (Daniel and Lees, 1993). Mild cognitive impairment (MCI) was diagnosed according to the Movement Disorder Society (MDS) criteria for MCI in PD (level I) (Litvan et al., 2011) using the Montreal Cognitive Assessment (MoCA; Nasreddine et al., 2005) and Spanish normative data (Ojeda et al., 2016). The disease severity was assessed according to the Hoehn and Yahr scale (H&Y) and the Unified Parkinson’s Disease Rating Scale (UPDRS; Fahn et al., 1987), assessed by a neurologist specialized in movement disorders and blind to the aim of the study. Patients in H & Y stage 4 and 5 were not included in this study. Other exclusion criteria included: (1) the existence of dementia diagnosed by a neurologist according to the MDS diagnostic criteria for PD dementia (Dubois et al., 2007); (2) the presence of other neurological or psychiatric disorders (i.e., traumatic brain injury or schizophrenia); and (3) the presence of visual hallucinations. Caregivers also completed the Neuropsychiatric Inventory Questionnaire (NPI-Q; Boada et al., 2002) to assess neuropsychiatric symptoms of the patients over the previous month. All patients were symptomatically stable, taking medication, and assessed during their “on” medication phase as reported by participants (see Table 1 for clinical details).

**TABLE 1:** Mean values (SD) for individuals with PD on demographic and clinical assessment carried out by a neurologist prior to the study.

Neurological assessment	Means (SD)	On-phase	Off-phase
Gender (male/female)	14/1		
Disease (years):			
Since the first symptoms appeared	6.87 (4.61)		
Since the diagnosis was made	5.56 (4.51)		
L-dopa (months of treatment)	44.40 (47.87)		
L-dopa (mg)	439.28 (268.13)		
LED (mg)	729.53 (298.23)		
UPDRS total score		25.60 (13.20)	24.60 (11.76)
Hoehn and Yahr scale		1.77 (0.37)	1.80 (0.36)

LED, total daily Levodopa equivalent dose; UPDRS, Unified Parkinson Disease Rating Scale (Fahn et al., 1987); H&Y, Hoehn, and Yahr scale. For each patient, the levodopa equivalent dose (LED) was calculated following the procedures of Tomlinson et al. (2010). LED represents the summary of antiparkinsonian drugs the patient receives and takes into account the intensity of different mediations (Tomlinson et al., 2010).

The control group was composed of 15 healthy adults (2 women, 66.9 ± 5.7), who were recruited through local advertisement, from a senior program of the University, or were University employees. None of the participants reported a history of neurological or psychiatric condition, alcohol or drug abuse, head trauma, or significant motor, visual or auditory deficits.

### Materials and procedure

The study was conducted in adherence with the Declaration of Helsinki (1991) and was approved by the Ethics Committee of the Balearic Islands. All participants provided informed consent before participation.

In this cross-sectional study, all potential participants were first interviewed for the purpose of screening based on the exclusion criteria described above. They underwent a neuropsychological and clinical assessment that was performed in a single session lasting around 90 min. To control for the presence of affective symptomatology, we used the Spanish version of the Patient Health Questionnaire for depression (PHQ-9; Diez-Quevedo et al., 2001) and the Generalized Anxiety Disorder questionnaire (GAD-7; Spitzer et al., 2006) to assess anxiety symptomatology. The evaluation of general cognitive profile was conducted with the Montreal Cognitive Assessment (MoCA; Nasreddine et al., 2005). In the context of a broader neuropsychological evaluation, we administered tests covering different cognitive processes (see Siquier and Andrés, 2021a,b, 2022). In the present work, attention/executive skills were examined with the forward and backward Digit Span Test (WAIS IV, Wechsler, 2012), phonemic (FAS, words beginning with F, A, and S for 1 min each) and semantic (animals) fluency tasks.

**FIGURE 1:**
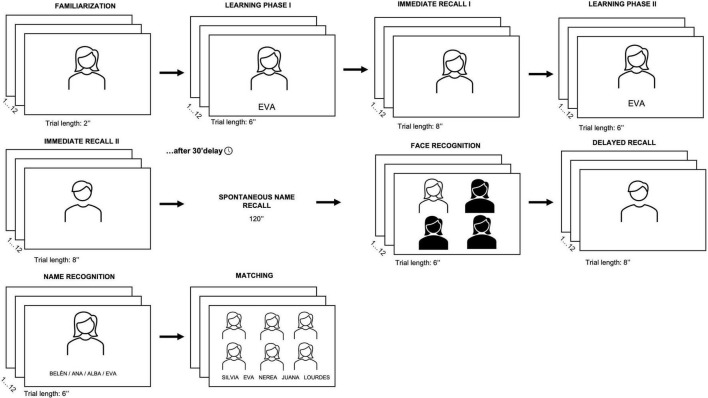
Outline of the extended and modified FNAME paradigm used. “The participants underwent two learning trials (Learning I and Learning II) to all the 12 face-name pairs. Following each trial, they were asked to provide the name associated with each face (Immediate recall I and II). Face-name pairs that were not remembered were shown again to the participant. After a 30-min delay, free recall, recognition and matching tasks followed. First, participants were asked to freely recall all names they learned within 2 min (Spontaneous recall). Next, they were asked to recognize the previously learned face out of 4 (Face Recognition). Then, they were again asked to say the name associated with each face (Delayed recall). After that, participants were asked to select the name associated with the face from among four items (Name recognition). Finally, participants were instructed to match the correct name to the corresponding face among the 6 presented (men and women were presented separately in the Matching phase). Participants’ responses were produced orally and recorded on a scoring sheet”.

The main measures for the purpose of the present study were the FNAME and the self-reported Memory failures of everyday questionnaire, MFE-28 (Montejo et al., 2012). The FNAME was administered as described in Flores Vazquez et al. (2021) study. Briefly summarized, the test included 12 unfamiliar face-name pairs to be memorized and recalled after 30 min (see Figure 1). The procedure began with the presentation of 12 unfamiliar faces displayed individually for 2 s each without names, followed by the presentation of the same faces associated with 12 names for 6 s each (Familiarization). Participants had to read the names out loud and try to learn each face-name pair (Learning phase I). Immediately after, the faces were presented one by one, for 8 s each, in a random (but always the same) order. Participants were asked to recall the name associated to each face in the earlier phase (Immediate cued-recall I). If the participant was unable to recall the name, the examiner provided the participant with the correct answer. This learning phase was then repeated once using a different ordering of the faces but with the same pairings. For the second time, the pictures were presented without names in a new random order (Learning phase II), after which participants were presented with the faces one by one and asked to recall the associated names (Immediate recall II). The number of correctly recalled pairs was recorded as an Immediate recall score (I & II; maximum score for these two recalls = 24). This was followed by a 30-min delayed recall test in which participants were given 2 min to free recall the names previously learned (without pictures; Spontaneous name recall). During this interval, other neuropsychological tests and clinical measures (i.e., QUIP-RS; SF-36; GAD-7; PHQ-9) were administered in the context of broader assessment (see Siquier and Andrés, 2021a,b, 2022). Subsequently, participants undertook a recognition task in which they were asked to discriminate between the previously learned face among three foils (Face Recognition). Next, the 12 learned faces were presented for 8 s each, without names, and participants were asked to recall out loud the associated name (Delayed recall). The 12 faces were then presented along with three new names of the same gender, and participants were asked to recognize the names that they had failed to retrieve in the delayed recall phase (Name Recognition). Finally, participants were shown a slide with the six women’s faces and, at the bottom of the screen, their names in a random order. They were asked to match the names to the faces. This task was then repeated with the male names and faces (Face-Name Matching). The maximum score for each individual test/variable was 12, and the maximum score for Total FNAME was 84.

Finally, participants completed the Spanish version of the Memory Failures of Everyday Questionnaire (MFE-28, Montejo et al., 2012), which served to assess memory forgetfulness and complaints. Participants rated 28 items on a 3- option response scale (0 = “never, rarely,” 1 = “occasionally, sometimes,” 2 = “frequently, almost always”) according to the frequency with which memory failures occur in their daily life. Thus, the total score ranged from 0 to 56, with higher values indicating a greater frequency of self-reported memory failures.

### Statistical analysis

Statistical analyses were performed using the SPSS software (version 26.0). Normality was checked with Shapiro-Wilk’s Test and homogeneity of variances with Levene’s Test (see Supplementary material). Mann-Whitney (*U*) tests were used to compare the PD and the healthy control groups in relation to sociodemographic, clinical and neuropsychological variables. Rank-biserial correlations (r*_*rb*_*) were derived as measures of effect sizes. Subsequently, FNAME performance was analyzed using analyses of covariance (ANCOVA) to control for potential group differences in key clinical or cognitive variables. *Post hoc* analyses were conducted using Tukey’s HSD. Effect sizes were measured using Partial η*^2^ and Cohen’s d*-values.

Associations between FNAME subscales, FAS, MoCA, MFE-28 and PHQ-9 scores were calculated with Spearman rho correlations on the total sample (with *p*-value adjustments for Bonferroni multiple comparisons) to explore any possible association between these clinical and neuropsychological variables.

## Results

Demographic characteristics and general cognitive performance of the sample are shown in Table 2. Individuals with PD and healthy controls did not differ on age or years of education. Both groups also showed similar global cognition (MoCA), attention, semantic fluency, working memory performance and visuospatial/executive skills measured with the Wechsler Adult Intelligence Scale’s (WAIS-IV) forward and backward digits test. Significant differences, however, were revealed in phonemic fluency (FAS), with the PD group performing significantly lower than the control group. The PD group also showed higher scores on PHQ-9 and MFE-28, reporting more depressive symptoms and daily memory problems than the control group. No significant differences were observed for anxiety (GAD-7).

**TABLE 2:** Demographic data and general cognitive performance (mean of raw scores and SDs) from individuals with PD and controls.

	PD	HC	U	*P*-value	*r*_*rb*_ ^§^
Age	67.40 (9.7)	68.90 (6.0)	103	0.71	–0.084
Education (years)	13.40 (4.6)	13.80 (3.7)	120	0.77	0.067
Depression (PHQ-9)*	6.06 (4.5)	2.26 (2.3)	182	0.004	0.618
Anxiety (GAD-7)	3.33 (2.9)	3.26 (3.4)	121	0.73	0.076
MFE-28	20.60 (10.4)	11.50 (3.7)	179	0.006	0.591
MoCA	26.50 (2.4)	27.40 (1.4)	95	0.47	–0.156
FAS (total of words)	34.53 (11.4)	49.13 (13.2)	41	0.003	–0.636
Semantic fluency (Animals)	16.66 (5.9)	20.60 (4.4)	75.5	0.12	–0.329
Forward DS	8.30 (2.3)	9.10 (3.3)	104	0.73	–0.076
Backward DS	6.73 (2.1)	7.40 (2.1)	90.5	0.36	–0.196

*P*-values and effect sizes^§^ are provided. PHQ-9, Patient Health Questionnaire-9; maximum score, 27, clinical threshold ≥ 10; GAD-7, 7-item anxiety scale; MFE, Memory Failures of Everyday questionnaire; MoCA maximum score, 30; cut-score ≥ 26; FAS, verbal phonemic fluency; forward and backward DS, Digit Span Test from the Wechsler Adult Intelligence Scale (WAIS)-III.

*Despite the significant differences between groups, depressive symptoms severity did not reach the clinical threshold (≥ 10) to meet criteria for depression.

Looking into individual performance, one patient was just below the cut off score for MCI according to the MoCA cut off score (24; Ojeda et al., 2016).

^§^Mann–Whitney test used. For the Mann–Whitney test, effect size is provided by the rank biserial correlation (*r_rb_*).

Recall performance on the FNAME is detailed in Table 3. As expected, performance was globally significantly lower in the PD group relative to the control group. The results revealed consistently lower free recall for individuals with PD at immediate, delayed recall, and matching with large and very large effect sizes (all *d* > 0.9). Concerning recognition, a smaller difference was observed between groups in name recognition (*d* = 0.44), and there were no differences in recognizing faces from learned face-name pairs (*p* = 0.173). This pattern of performance suggests that individuals with PD showed greater difficulties in recalling names compared to recognizing faces.

**TABLE 3:** Means and standard deviations (in brackets) for recall and recognition performance (raw scores) on the FNAME.

Type of recall	PD	HC	*F*	*p before*	*F*	*p after*
Immediate recall I	2.5 (2.8)	5.1 (2.7)	2.1	0.15	N/A	N/A
Immediate recall II	4.6 (3.0)	8.7 (2.4)	5.7	0.02	N/A	N/A
Total IR (I + II)	7.2 (5.6)	13.8 (4.7)	4.2	0.04	N/A	N/A
Spontaneous name recall	5.3 (2.1)	7.8 (1.5)	3.6	0.06	0.12	0.73
Face recognition	11.0 (1.6)	11.8 (0.41)	0.98	0.33	0.31	0.58
Delayed recall	3.8 (3.4)	7.8 (2.6)	5.6	0.026	1.2	0.29
Name recognition	9.8 (2.27)	11.46 (0.91)	6.7	0.016	2.6	0.11
Matching score	5.8 (3.0)	10.8 (1.8)	13.9	0.001	8.3	0.008
Total score	43.0 (16.2)	63.7 (9.6)	7.6	0.010	N/A	N/A

*P*-values resulting from ANCOVA controlling for depression and FAS are provided. *P*-values are provided before and after control for initial learning (Total immediate recall) in addition to FAS and depression scores. PD, Parkinson disease; HC, healthy controls; FNAME, Face–Name Associative Memory Exam; Total IR, Total immediate recall; *p before*, *p*-value before controlling for depression (PHQ-9), and phonemic fluency (FAS) scores (no immediate total recall), *p after*, *p*-value after control for immediate total recall, PHQ-9 and FAS; N/A, non-applicable. When controlling for the initial learning trials, only the differences in matching remained significant.

**FIGURE 2:**
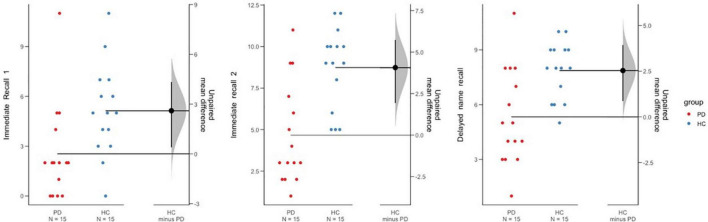
The mean difference in recall 1, recall 2, and delayed name recall between groups is shown in the Gardner- Altman estimation plots. Groups are plotted on the left axes. The right panel is a one-dimensional plot (zero is centered on the mean of one of the groups), where the mean difference is plotted on a floating axis as a bootstrap sampling distribution. The mean differences are depicted as a dot. The 95% CI are indicated by the ends of the vertical error bars.

Recall subscores were analyzed using a 2 (group) × 3 (Immediate Recall I, Immediate Recall II, Delayed Recall) ANCOVA controlling for depression and phonemic fluency (FAS). This analysis revealed a significant effect of group [*F*(1, 26) = 4.39; *p* = 0.046, η*_*p*_*^2^ = 0.145] and a significant effect of type of recall [*F*(1, 26) = 13.45; *p* = 0.001, η*_*p*_*^2^ = 0.341]. Unsurprisingly, more items were recalled at Immediate recall II than at Immediate recall I [*t*_(29)_ = –7.549, *p* < 0.001, *d* = 1.37] (see Figure 2). The number of items freely recalled were similar after the 30-min delay [*t*_(29)_ = 0.351, *p* = 0.728, *d* = 0.06]. No interaction was found between variables.

The effect of the covariates was not significant: *F*(1, 26) = 2.11, *p* = 0.157, η*_*p*_*^2^ = 0.075, for FAS, and *F*(1, 26) = 0.022, *p* = 0.88, η*_*p*_*^2^ = 0.001, for depression.

We reanalyzed the data by controlling for the differences observed in the acquisition of information (using total immediate recall as covariate) in order to assess the extent to which an encoding deficit would account for memory differences observed at recall (see Chiaravalloti et al., 2014; Siquier and Andrés, 2021a). As can be seen in Table 3, significant differences between groups disappeared after controlling for initial learning (this is, total immediate recall), except for the face-name matching score, indicating that the deficit observed on matching in PD could not be explained by a deficit at encoding.

### Correlational analyses

Correlations are presented in Table 4. After applying Bonferroni correction (adjusted α = 0.05/11 = 0.0045), the unique correlation that remained significant with subjective memory, measured by the MFE-28, was the association with the face-name Matching score (*r* = –0.581, *p* < 0.001).

**TABLE 4:** Correlations between FNAME, MoCA, and FAS performance, MFE, and PHQ-9 scores.

	MoCA	Total IR	SNR	Recog I	DR	Recog II	Matching	Total score	PHQ-9	FAS
MFE	–0.412	–0.377	–0.322	–0.377	–0.486	–0.248	−0.581*	–0.420	0.396	–0.399
MoCA		0.350	0.403	0.110	0.393	0.178	0.431	0.363	–0.203	0.344
Total IR			0.873*	0.321	0.827*	0.604*	0.810*	0.940*	–0.414	0.538
SPR				0.435	0.879*	0.691*	0.801*	0.929*	–0.484	0.416
Recog I					0.314	0.299	0.275	0.378	–0.272	0.160
DR						0.690*	0.928*	0.948*	–0.455	0.419
Recog II							0.658*	0.748*	–0.100	0.059
Matching								0.910	–0.485	0.534*
Total score									–0.433	0.493
PHQ-9										−0.556*

*p < 0.001. MFE Memory Failures of Everyday Questionnaire; MoCA, Montreal Cognitive Assessment; Total IR, Total immediate recall; SNR, Spontaneous Name Recall; Recog, Recognition; DR, Delayed Recall; FNAME, Face–Name Associative Memory Exam; PHQ-9, Patient Health Questionnaire-9; FAS, phonemic fluency test.

*After applying Bonferroni correction (adjusted α = 0.05/11 = 0.0045), the unique correlation that remained significant with MFE was the association with the FNAME Matching score.

## Discussion

Memory complaints are frequent in PD (Erro et al., 2014; Copeland et al., 2016; Mills et al., 2016). However, the clinical value of these complaints and the source of these deficits is not yet completely understood. The present study provides insights into the pattern of memory impairment in PD and the relationship between neuropsychological objective outcomes and subjective experience of memory difficulties in everyday life.

Memory abilities were assessed by an adapted computerized version of the Face-Name Associative Memory Exam (FNAME) (Enriquez-Geppert et al., 2021; Flores Vazquez et al., 2021; Flores-Vazquez et al., in press) and by the self-rating Memory Failures of Everyday questionnaire (MFE; Montejo et al., 2012). The FNAME paradigm has the advantage of tapping more ecologically relevant memory skills (Loewenstein et al., 2018) than other memory tests. Aside from the higher ecological validity, due to the arbitrary and unique association between faces and names, the processes involved in the FNAME are more demanding than verbal recognition or other visual memory tasks (Werheid and Clare, 2007). Thus, it provides highly sensitive indices of episodic memory performance. In this vein, higher face-name task difficulty has been shown to be associated with greater neural activity in the bilateral prefrontal cortex (Yu et al., 2021). Also, compared to pen-and-paper tests, computerized tests reduce the risk of administration and scoring errors and ensure a more standardized procedure. In addition, as this paradigm does not include complex language requirements, it may be particularly suitable to examine individuals with low educational levels or verbal difficulties.

The results showed significantly lower performance in the PD group than in the healthy group on the FNAME task, except for face recognition. These findings indicate that the memory deficit observed in individuals with PD relates to a difficulty in freely retrieving the material. In that sense, our results are in line with the evidence showing retrieval problems in these patients. Growing evidence, however, suggests that at least part of the memory deficit observed in PD also results from poor encoding as a consequence of hippocampal alterations (Brønnick et al., 2011; Chiaravalloti et al., 2014). As it is the case with the Free and Cued Selective reminding test (see Siquier and Andrés, 2021a), an advantage of the FNAME task is that it enables to equate groups for encoding strategies, allowing therefore to rule out the contribution of learning differences to retrieval deficits. Once the groups were equated with respect to encoding, the effect of group on delayed free recall disappeared. These findings suggest that an encoding dysfunction relating to a deficient use of learning strategies, may also account for at least part of the memory deficits observed in individuals with PD.

Furthermore, the recently added matching subscore (see Enriquez-Geppert et al., 2021; Flores Vazquez et al., 2021) reinforces the evidence that associative memory is also compromised in PD. Matching consisted in pointing to the faces and names that belonged together among all pairs (men and women presented separately). This is a more genuine exercise of associative memory than the other subtests and involves binding processes (referred as the generation of associative links between independent items or between items and a context). Our results suggested that the face-name matching score was the most sensitive measure to distinguish between PD and control groups. First, differences between groups remained significant despite controlling for initial learning. Second, the matching score showed the largest correlation with self-reported memory failures. It may therefore serve as a possible indicator of subtle memory deficits. Overall, our findings suggest that associative face-name memory is a fruitful neuropsychological domain to characterize the PD memory profile and capture the nature of their daily life memory problems. Binding deficits have been thoroughly investigated in Alzheimer’s disease (Della Sala et al., 2012; Liang et al., 2016) and aging (Enriquez-Geppert et al., 2021; Flores Vazquez et al., 2021), but, to date, studies of associative memory in PD are scarce (Cohn et al., 2016; Bezdicek et al., 2019). Yet this study suggests that binding may be a memory component affected early in PD.

Moreover, binding has been linked to the hippocampus, supporting the idea of a memory impairment due to the hippocampal neurodegeneration also frequently observed in PD (Junqué et al., 2005; Bezdicek et al., 2019) in addition to the fronto-striatal disruption. In that sense, the FNAME matching score might also emerge as an indirect measure of hippocampal dysfunction in PD. Future research adopting measures of the processes tapped by FNAME and integrating functional neuroimaging techniques would be useful to clarify the potential relationship between the fronto-striatal and hippocampal subfields in relation to specific memory disruptions in PD.

Looking at the impact of memory complaints in daily life, individuals with PD indicated more subjective memory difficulties compared to healthy controls. These results are consistent with some previous studies (Erro et al., 2014; Lehrner et al., 2014). Yet, not all previous studies observed greater memory complaints reported by these patients. For example, Dupouy et al. (2018) did not find any significant subjective cognitive complaint in the memory domain, nor in the visuospatial domain, evaluated by a visual analog scale (VAS). Importantly, however, they observed subjective cognitive complaints in executive functions, language, and attention. However, these authors did not report objective measures of cognitive performance. Methodological factors, such as the considerable variability in the measures used to assess complaints hamper the comparison of studies. One possible reason for this inconsistency may lie with the lack of a standardized measure of subjective cognitive complaints. Subjective cognitive decline being a criterion recommended by the MDSs task force (Litvan et al., 2012) for the diagnosis of MCI in PD, these findings reinforce the necessity to establish reliable tools to measure the self-appraisal of cognitive difficulties in PD.

Some limitations of the present study should be pointed out. First, the difficulty in recruiting participants with PD that fulfilled our strict inclusion and exclusion criteria resulted in a relatively small sample. We cannot exclude, however, that this novel pilot study was underpowered to detect some effects of small size. Second, inherent to its cross-sectional design, the potential role of associative memory deficits as a predictor of SMC in PD can only be hypothesized, requiring further longitudinal studies. Third, despite the fact that PD is 1.5 times more common in men than in women (Elbaz et al., 2016), our sample only included one woman. Thus, further studies including larger samples should also include more women to improve external validity and further investigate the pattern of memory deficits and the relationship with the subjective experience of daily life memory difficulties in PD.

Fourth, we were interested in the person’s objective and subjective experience of memory daily difficulties, without considering caregiver’s perception. Asking caregivers to comment on patients’ memory performance would have answered a different, although interesting, question. Future studies should include a comparison between patients and caregivers’ perception to gather an accurate picture of memory failures and their appraisal in patients with PD.

Finally, MFE-28 was originally developed to detect cognitive difficulties typically present in AD. However, up to date, there are no available guidelines for suitable assessment tools to examine SMC in PD (Kjeldsen and Damholdt, 2019). Given the diversity and the current lack of validated tools, there is a need to develop more specific instruments sensitive to early subjective cognitive complaints in PD.

Keeping the above-mentioned limitations in mind, the present study highlights the relevance of using more challenging and ecological tests to detect subtle memory difficulties in PD. The integration of binding measures might contribute to capture more accurately different components of memory function in PD. In this context, our results demonstrate that associative memory, measured by the FNAME could potentially emerge as an effective neuropsychological tool to assess different memory deficits in PD.

## Data availability statement

The raw data supporting the conclusions of this article will be made available by the authors, without undue reservation.

## Ethics statement

The studies involving human participants were reviewed and approved by the Ethics Committee of the Balearic Islands. The patients/participants provided their written informed consent to participate in this study.

## Author contributions

AS and PA: conceptualization, investigation, visualization, and writing—review and editing. AS: data curation, formal analysis, and writing—original draft. PA: project administration, supervision, and funding acquisition. Both authors have read and agreed to the published version of the manuscript.

## Footnotes

1 One previous study (Kormas et al., 2019), administered the FNAME-12 to PD patients, but only a global comparison between patients was carried out, and, importantly, there was no comparison with healthy control participants, thereby limiting the interpretation of the results.
