# Supplementary material for: Face name matching and memory complaints in Parkinson’s disease
Source: Front Psychol. 2022 Nov 14;13:1051488. doi: 10.3389/fpsyg.2022.1051488 (PMC9702071; doi:10.3389/fpsyg.2022.1051488)
Supplement: Supplementary file 1 [file Table_1.DOCX]

Supplementary Material

| **Test of Normality (Shapiro-Wilk)** | | | | | | | |
| --- | --- | --- | --- | --- | --- | --- | --- |
|  | |  | | **W** | | ***p*** | |
| Age |  | PD |  | 0.949 |  | 0.506 |  |
|  |  | HC |  | 0.918 |  | 0.181 |  |
| Education |  | PD |  | 0.749 |  | 0.001 |  |
|  |  | HC |  | 0.881 |  | 0.050 |  |
| GAD-7 |  | PD |  | 0.884 |  | 0.054 |  |
|  |  | HC |  | 0.841 |  | 0.013 |  |
| PHQ-9 |  | PD |  | 0.790 |  | 0.003 |  |
|  |  | HC |  | 0.869 |  | 0.032 |  |
| MFE |  | PD |  | 0.981 |  | 0.974 |  |
|  |  | HC |  | 0.888 |  | 0.062 |  |
| MOCA |  | PD |  | 0.858 |  | 0.022 |  |
|  |  | HC |  | 0.945 |  | 0.446 |  |
| FAS |  | PD |  | 0.975 |  | 0.919 |  |
|  |  | HC |  | 0.906 |  | 0.119 |  |
| Animals |  | PD |  | 0.938 |  | 0.354 |  |
|  |  | HC |  | 0.874 |  | 0.039 |  |
| Forward DS |  | PD |  | 0.870 |  | 0.034 |  |
|  |  | HC |  | 0.902 |  | 0.104 |  |
| Backward DS |  | PD |  | 0.875 |  | 0.039 |  |
|  |  | HC |  | 0.896 |  | 0.084 |  |
| FNAME Total |  | PD |  | 0.928 |  | 0.256 |  |
|  |  | HC |  | 0.939 |  | 0.364 |  |
|  | | | | | | | |

**Test of Equality of Variances (Levene's)**

| **Variables** | | **F** | | ***p*** | |  |
| --- | --- | --- | --- | --- | --- | --- |
| Age |  | 2.616 |  |  | 0.117 |  |
| Education |  | 4.470 |  |  | 0.044 |  |
| GAD-7 |  | 1.483 |  |  | 0.233 |  |
| PHQ-9 |  | 1.383 |  |  | 0.249 |  |
| MFE |  | 12.516 |  |  | 0.001 |  |
| MOCA |  | 4.921 |  |  | 0.035 |  |
| FAS |  | 0.026 |  |  | 0.872 |  |
| Animals |  | 0.912 |  |  | 0.348 |  |
| Forward DS |  | 2.998 |  |  | 0.094 |  |
| Backward DS |  | 0.008 |  |  | 0.930 |  |
| Total FNAME |  | 3.787 |  |  | 0.062 |  |

**Abbreviations.**

GAD-7: 7-item anxiety scale; PHQ-9: Patient Health Questionnaire-9; MFE: Memory Failures of Everyday questionnaire; MoCA maximum; FAS: verbal phonemic fluency; Forward and backward DS: Digit Span Test from the Wechsler Adult Intelligence Scale (WAIS)-III
